# Person-Centered Trajectories of Psychopathology From Early Childhood to Late Adolescence

**DOI:** 10.1001/jamanetworkopen.2022.9601

**Published:** 2022-05-10

**Authors:** Colm Healy, Ross Brannigan, Niamh Dooley, Lorna Staines, Helen Keeley, Robert Whelan, Mary Clarke, Stanley Zammit, Ian Kelleher, Mary Cannon

**Affiliations:** 1 Department of Psychiatry, Royal College of Surgeons in Ireland, Dublin, Ireland; 2 Department of Sociology, Trinity College Dublin, Dublin, Ireland; 3 Child and Adolescent Mental Health Services North Cork, Health Service Executive, Ireland; 4 Department of Psychology, Trinity College Dublin, Dublin, Ireland; 5 Department of Psychology, Royal College of Surgeons in Ireland, Dublin, Ireland; 6 Centre for Academic Mental Health, Bristol Medical School, University of Bristol, Bristol, United Kingdom; 7 School of Medicine, University College Dublin, Dublin, Ireland; 8 Lucena Clinic, St John of God Community Mental Health Services, Dublin, Ireland; 9 Department of Psychiatry, Beaumont Hospital, Dublin, Ireland

## Abstract

**Importance:**

The understanding of the development of psychopathology has been hampered by a reliance on cross-sectional data and symptom- or disorder-centered methods. Person-centered methods can accommodate both the problems of comorbidity and the movement between different psychopathological states at different phases of development.

**Objective:**

To examine the profiles and map the trajectories of psychopathology from early childhood to late adolescence.

**Design, Setting, and Participants:**

This cohort study used 2 longitudinal nationally representative community-based cohorts from the Growing Up in Ireland study covering developmental periods from early childhood to late adolescence. Data in this investigation came from children and their families who participated in all waves of cohorts recruited in 2008 (children ages 3, 5, and 9 years) and 1998 (adolescents ages 9, 13, and 17 or 18 years). Both samples were weighted to account for representation and attrition. Latent transition analyses were used to map the trajectories of psychopathology. Data were analyzed between October 2020 and September 2021.

**Main Outcomes and Measures:**

Psychopathology was measured using the Strengths and Difficulties Questionnaire at all ages in both samples.

**Results:**

A total of 13 546 individuals were included in the analyses. In the child cohort, mean (SD) age was 3.0 [0.01] years; 3852 (51.3%) were male participants. In the adolescent cohort, mean age was 9.0 (0.1) years; 3082 (51.0%) were male participants. Four profiles were identified in both cohorts that could be broadly labeled as no psychopathology (incidence range, 60%-70%), high psychopathology (incidence range, 3%-5%), externalizing problems (incidence range, 15%-25%), and internalizing problems (incidence range, 7%-12%). Transition between the profiles was common in both cohorts, with 3649 of 7507 participants (48.6%) in the child cohort and 2661 of 6039 participants (44.1%) in the adolescent cohort moving into 1 of the 3 psychopathology profiles at some point in development. Transition to the high psychopathology profile was most often preceded by externalizing problems. Approximately 3% to 4% of the sample had persistent psychopathology (child cohort, 203 participants [2.7%]; adolescent cohort, 216 participants [3.6%]). All psychopathology profiles were more common in boys in early life but, by late adolescence, girls were more likely to have internalizing problems. In a cross-cohort comparison at age 9, there were differences in the sex distributions of the profiles between the samples.

**Conclusions and Relevance:**

Using person-centered methods, this study demonstrated that from early life young peoples’ experience of psychopathology is dynamic—they can move between different mental health problems; for most children, these problems are transient, but a small proportion (fewer than 5%) have persistent difficulties. In the context of finite resources, optimizing care requires the early identification of those with persistent phenomena.

## Key Points

**Question:**

How can the profiles and trajectories of psychopathology from early life (age 3 years) to late adolescence (age 17 years) be mapped with person-centered methods to accommodate problems of comorbidity and movement between different phases of development?

**Findings:**

This cohort study of 2 nationally representative cohorts of Irish children developed 4 distinct developmental profiles for person-centered psychopathological trajectories. Over 44% of children transitioned into 1 of the 3 psychopathological profiles during development, with less than 5% of the sample experiencing persistent psychopathology.

**Meaning:**

These results suggest that psychopathology is common in childhood but mostly limited in duration and that optimizing care in the context of finite resources may require the early identification of those with persistent phenomena.

## Teaser

This cohort study of 2 longitudinal cohorts of Irish children and adolescents develops a schema of person-centered psychopathological profiles based on evaluative questionnaire data.

## Introduction

Mental disorders are among the leading causes of lifetime disability^1,2,3^ and their prevention has been proposed as a major challenge of this century.^4,5^ Caspi and colleagues^6^ have proposed that “our understanding of mental disorders is hampered by a reliance on cross-sectional data.” It has been demonstrated, using cohort samples^6^ as well as retrospective survey^7^ and registry data,^8^ that simultaneous and sequential comorbidities are evident within and across diagnostic clusters, and that individuals move between diagnoses over their lifespan.

The problems of comorbidity are compounded in early life, when psychiatric symptoms are fluid phenomena that can change with development.^9,10^ Few studies have attempted to map the fluidity of psychopathology across early development and most use symptom-centered approaches, eg, changes in the severity of specific symptoms across time.^11,12,13^ A person-centered trajectory approach allows for data to reflect that individuals have multiple different symptoms at each time point and to map changes in these symptoms across time. This aligns closely with both the observed reports of simultaneous and sequential comorbidity^6,7,8^ and the dynamic and nondeterministic nature of psychopathology in youth.^10^ The use of these approaches assumes that there are clusters of psychiatric symptoms in the population but that individuals within these clusters may change with development. A review found 23 eligible studies using latent class analysis, but only 7 investigated a broad range of symptoms occurring simultaneously.^14^ Latent transition analysis (ie, transition between the classes) has rarely been investigated^15,16^ and aligns with the dynamic nature of psychopathology in youth.^10^ Moreover, there are well-established differences in biological sex between the different mental disorders. It is unclear if the differences in biological sex also affect longitudinal trajectories.

Using data from 2 longitudinal cohorts and latent profile transition analysis (LPTA), we aimed to map individuals’ trajectories of psychopathology from early childhood (age 3 years) to late adolescence (age 18 years). First, we examined the symptom clustering and the percentage of the population within these profiles at multiple points across development. Second, we mapped the trajectories between the different profiles. We ran supplementary investigations examining whether there were sex differences in the profiles and generational differences based on a comparison of results between cohorts at age 9 years.

## Methods

### Participants

We used data from the Growing Up in Ireland study (GUI), which comprises 2 large nationally representative cohorts spanning from infancy to late adolescence (the 1998 cohort and 2008 cohort). The 2008 cohort covers the period of development from ages 9 months to 9 years (7507 participants); for the purpose of this investigation the 2008 cohort is referred to as the *child sample*. The 1998 cohort covers the period from ages 9 years to 17 or 18 years (6039 participants); for the purpose of this investigation the 2008 cohort is referred to as the *adolescent sample*. (For further information on the sampling method, representative weighting, attrition, and low to follow-up adjustment see eMethods and eTable 1 in the Supplement). Full information on these cohorts is available in the studies from Layte and McCrory^17^ and Williams et al.^18^ Informed consent was obtained from the parent or guardian of the participating child and assent was obtained from the child themselves at each wave of the studies by the Growing Up in Ireland study team. Ethical approval for the GUI study was granted by the research ethics committee of the Health Research Board in Ireland and Royal College of Surgeons in Ireland, and approval for this study was granted by the research ethics committee of the Royal College of Surgeons in Ireland.

### Demographics and Psychopathology Measurement

Demographic variables reported to characterize the sample included age, sex, handedness, and nationality (Irish or non-Irish) of the child, the nationality of the primary caregiver, urbanicity (urban or rural), and highest level of education of the primary caregiver (Table 1). Nationality was used instead of race or ethnicity as this information was available for the participating child and the primary care giver in both cohorts.

**Table 1.** Characteristics of the Child and Adolescent Samples^a^

Characteristics	Child sample, No. (%) (n = 7507)	Adolescent sample, No. (%) (n = 6039)
Age, mean (SD), y	3.0 (0.01)	9.0 (0.1)
Sex		
Boys	3852 (51.3)	3082 (51.0)
Girls	3655 (48.7)	2957 (49.0)
Left handedness	965 (12.9)^b^	772 (12.8)
Primary caregiver non-Irish nationality	1460 (19.5)^c^	898 (14.9)
Child non-Irish nationality	71 (0.9)^c^	632 (10.5)
Living in urban area	3297 (44.2)	2650 (44.0)
Primary caregiver highest level of education^d^		
None/primary	177 (2.4)^c^	339 (5.6)
Lower secondary	1033 (13.8)^c^	1421 (23.5)
High secondary, technical vocational, or upper secondary	2465 (32.9)	2239 (37.1)
Non-degree accreditation	1586 (21.2)^c^	994 (16.5)
Primary degree	1237 (16.5)^c^	675 (11.2)
Postgraduate	997 (13.3)^c^	370 (6.1)
SDQ subscales wave 1, mean (SD) score^e^		
Emotional problems	1.4 (1.4)	2.1 (2.1)
Peer problems	1.2 (1.4)	1.2 (1.5)
Conduct problems	2.2 (1.8)	1.3 (1.5)
Hyperactivity/attention	3.3 (2.2)	3.2 (2.5)
SDQ subscales wave 2, mean (SD) scores^f^		
Emotional problems	1.6 (1.7)	1.9 (2.0)
Peer problems	1.0 (1.4)	1.2 (1.5)
Conduct problems	1.5 (1.5)	1.2 (1.5)
Hyperactivity/attention	3.4 (2.5)	2.8 (2.7)
SDQ subscales wave 3, mean (SD) scores^g^		
Emotional problems	2.1 (2.1)	2.0 (2.1)
Peer problems	1.1 (1.6)	1.4 (1.5)
Conduct problems	1.2 (1.4)	1.0 (1.3)
Hyperactivity/attention	3.2 (2.6)	3.4 (2.3)

Abbreviation: SDQ, Strengths and Difficulties Questionnaire.

^a^ Both sets of results are weighted to match the Irish 2006 census and to account for attrition. All data are taken from the first wave when the strengths and difficulties questionnaire was administered (child cohort: wave 1, age 9 years; infant cohort: wave 2, age 3 years).

^b^ Asked at wave 3 (age 5 years).

^c^ Indicates a *P* value <.05.

^d^ Collected at first wave of SDQ collection.

^e^ Ages 3 and 9 years.

^f^ Ages 5 and 13 years.

^g^ Ages 9 and 17 years.

Psychopathology was measured using the 4 problem subscales of the Strengths and Difficulties Questionnaire (SDQ): emotional problems, peer problems, conduct problems, and hyperactivity.^19,20,21^ It was measured at ages 3, 5, 9 years in the child sample and ages 9, 13, and 17 years in the adolescent sample. In both cohorts and at all ages all data was provided by the primary caregiver (eTables 2 and 3 in the Supplement).

### Statistical Analysis

Analyses were carried out in Mplus version 8^22^ and Stata version 15 (StataCorp). Analyses were conducted using latent profile transition analysis following guidelines from Masyn.^23^ Both cohorts were weighted to ensure representation and to account for attrition (eMethods in the Supplement). Global model fit criteria (bayesian information criteria [BIC], adjusted BIC, and Akaike information criterion) and profile interpretability were used to estimate the optimum number of profiles.^24^ Time varying and time invariant solutions were compared using log likelihood ratio tests and BIC. Multinomial logistic regression was conducted to examine potential differences in sex distribution between the profiles, transitions, persistence of profiles, and cohorts. These analyses were adjusted for demographic difference between the cohorts, and results are reported as incidence risk ratios (IRR) (eMethods in the Supplement). The threshold of significance was set at α = .05.

## Results

### Descriptive Statistics

Data were available for 7507 children from the child sample who participated in the 3 waves of the study (ages 3, 5, and 9 years) and for 6039 children from the adolescent sample (ages 9, 13, and 17 or 18 years) (Table 1). In the child cohort, the mean (SD) age was 3.0 [0.01] years and 3852 (51.3%) were male participants. In the adolescent cohort, mean age was 9.0 (0.1) years and 3082 (51.0%) were male participants. The adolescent sample had a significantly higher percentage of non-Irish children (632 participants [10.5%] vs 71 participants [0.9%]). The child sample had a higher percentage of caregivers with higher primary or postgraduate degrees (postgraduate degree, 997 [13.3%] vs 370 [6.1%]) and a higher percentage of caregivers who were non-Irish (1460 [19.5%] vs 898 [14.9%]).

#### Latent Profile Analysis

We fitted a series of cross-sectional latent profile analysis (LPA) models for each age starting with a 2-profile model. The number of profiles were sequentially increased from 1 to 8. There was continuously improving fit indices with increasing profiles at all ages (eTable 4, eTable 5, eFigure 1 in the Supplement). The diminishing gains in the fit indices indicated that a 4-profile model was the best fit to the data such that 4 distinct symptom clusters existed at each time point, for both cohorts. For completeness, we also compared the 3-profile and 5-profile solutions for the latent profile transition analyses with the 4-profile solution.

#### Latent Profile Transition Analysis

**Figure 1.**
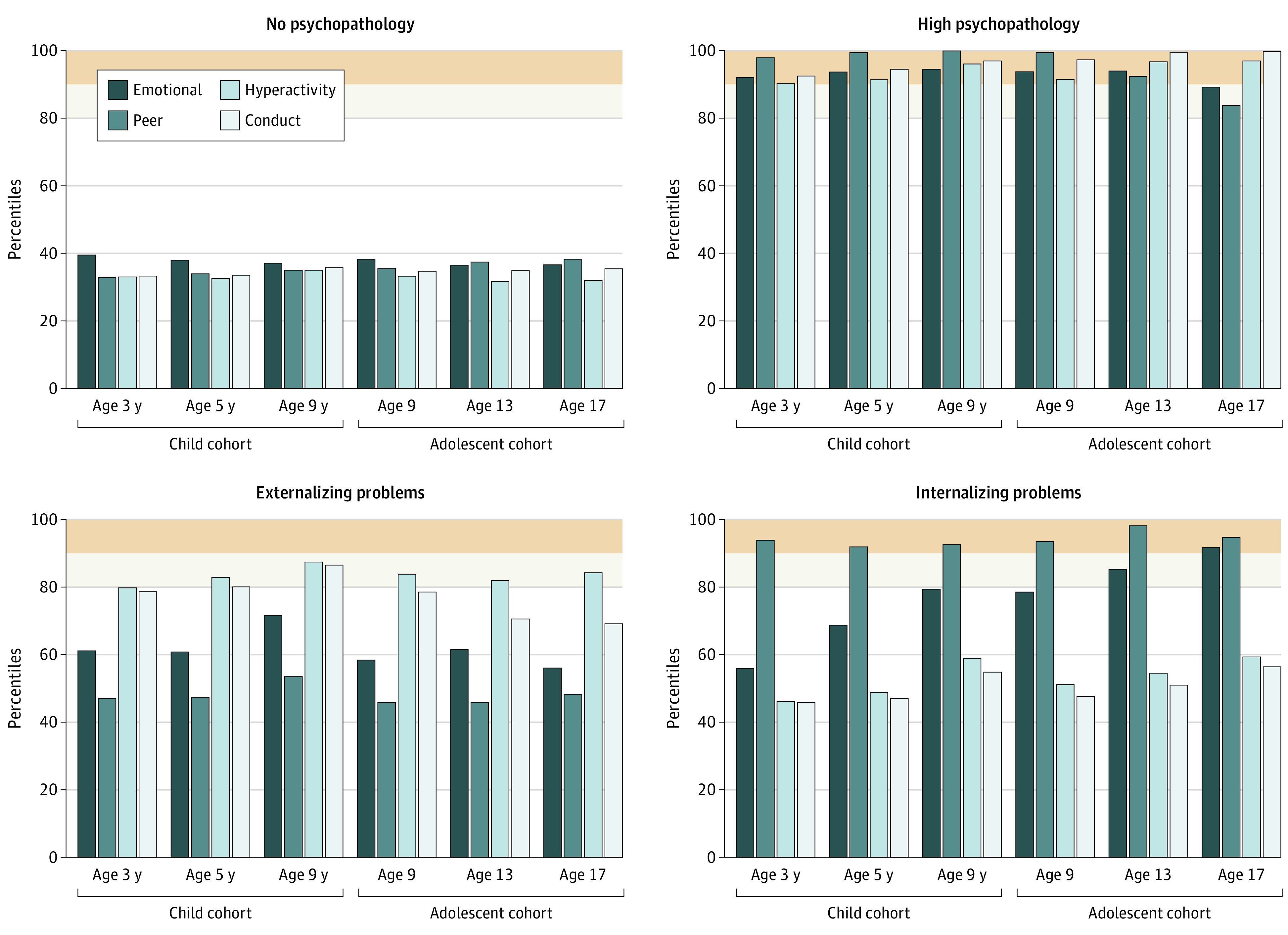
The Cross-sectional Percentile Scores on the Strengths and Difficulties Questionnaire Subscales in Each Profile at 3 Time Points of the Child and Adolescent Cohorts The beige band in each graph denotes the classic abnormal threshold (ie, >90th percentile); off-white band, an elevated but subclinical level (80th-90th percentile).

Based on the information gathered in the LPA we examined the LPTA models. Similarly, while we observed continuously decreasing fit, there was an elbow with the 4-profile model across indices in both cohorts (eMethods and eFigure 2 in the Supplement). We also considered the 3-profile and 5-profile solution relative to the 4-profile solution as well as time varying and time invariant solutions (eAppendix in the Supplement). Briefly, the 4-profile solution had better global model fit (base on Bayesian information criteria metrics and diminishing gains) and clearer profile distinguishing features relative to the 3-profile or 5-profile solution. In both cohorts the time vary model had significantly better model fit (child sample, χ^2^_32_ = 717.6, *P* < .001; adolescent sample, χ^2^_32_ = 201.9, *P* < .001). The full rationale for the selection of a 4-profile time-varying solution are presented in supplementary material (eAppendix in the Supplement). Thus, the final model in both cohorts (from ages 3 to 9 years and 9 to 17 years) over 3 waves consisted of 4 profiles with 64 potential transition pathways between the profiles. In both cohorts, we examined the mean and standard error of each of the SDQ subscale scores in each of the profiles (Figure 1). There were similar profiles observed in both cohorts. Based on the profiles produced by LPTA the groups were labeled: no psychopathology, externalizing problems, internalizing problems, and high psychopathology.

### Profiles

#### No Psychopathology

This profile was characterized by low scores (ie, 30th to 40th percentile) on all SDQ subscales at each wave in both sample groups. It accounted for approximately 60% to 70% of the participants in each sample at any point in time (eg, 4487 children [59.8%] in child cohort wave 1), with similar percentages for both sexes.

#### High Psychopathology

This profile was characterized by significant (above 90th percentile) scores across all SDQ problem subscales at almost all time points (Figure 1). This profile accounted for approximately 3% to 5% of the sample at all ages (eg, 346 children [4.6%] in child cohort wave 1). There was a higher proportion of boys in the high psychopathology profile at all ages of the child sample (age 3: IRR, 1.76; 95% CI, 1.41-2.21; *P* < .001; age 5: IRR, 2.02; 95% CI, 1.63-2.51; *P* < .001; age 9: IRR, 2.34; 95% CI, 1.79-3.08; *P* < .001) but only at age 13 years in the adolescent sample (IRR, 1.30; 95% CI, 1.02-1.65; *P* = .04) relative to the no psychopathology profile.

#### Externalizing Problems

This profile was characterized by elevated scores (70th to 90th percentile) in the hyperactivity and conduct subscales but average (45th to 72nd percentile) scores in the emotional and peer problems subscales. This profile accounted for approximately 15% to 25% of the population at all ages in both cohorts (eg, 1804 children [24.0%] in child cohort wave 1) and there was a greater proportion of boys in this profile at all ages relative to the no psychopathology profile in both child (age 3: IRR, 1.32; 95% CI, 1.18-1.47; *P* < .001; age 5: IRR, 1.55; 95% CI, 1.38-1.73; *P* < .001; age 9: IRR, 1.80; 95% CI, 1.58-2.05; *P* < .001) and adolescent samples (age 9: IRR, 1.34; 95% CI, 1.18-1.52; *P* < .001; age 13: IRR, 1.22; 95% CI, 1.08-1.39; *P* < .001; age 17: IRR, 1.38; 95% CI, 1.21-1.57; *P* < .001).

#### Internalizing Problems

This profile was characterized by significant (above 90th percentile) scores in the peer problems subscale and increasing levels of emotional problems across development (ie, 58th percentile at age 3 years to the 80th percentile at 9 years; in the adolescent sample, from the 79th percentile at age 9 years to the 93rd percentile at 17 years). Average (45th to 60th percentile) scores were reported for participants in the hyperactivity and conduct subscales. This profile accounted for approximately 7% to 12% of participants at all ages in both cohorts (eg, 869 children [11.6%] in the child cohort wave 1). In early life, there was a higher proportion of boys in this profile (age 3: IRR, 1.51; 95% CI, 1.30-1.75; *P* < .001; age 5: IRR, 1.18; 95% CI, 1.01-1.37; *P* = .04; age 9: IRR, 1.30; 95% CI, 1.12-1.53; *P* = .001); however, by late adolescence there were more girls in this profile (age 17: IRR, 1.76; 95% CI, 1.47-2.12; *P* < .001) relative to the no psychopathology profile.

### Trajectories Between the Profiles

**Figure 2.**
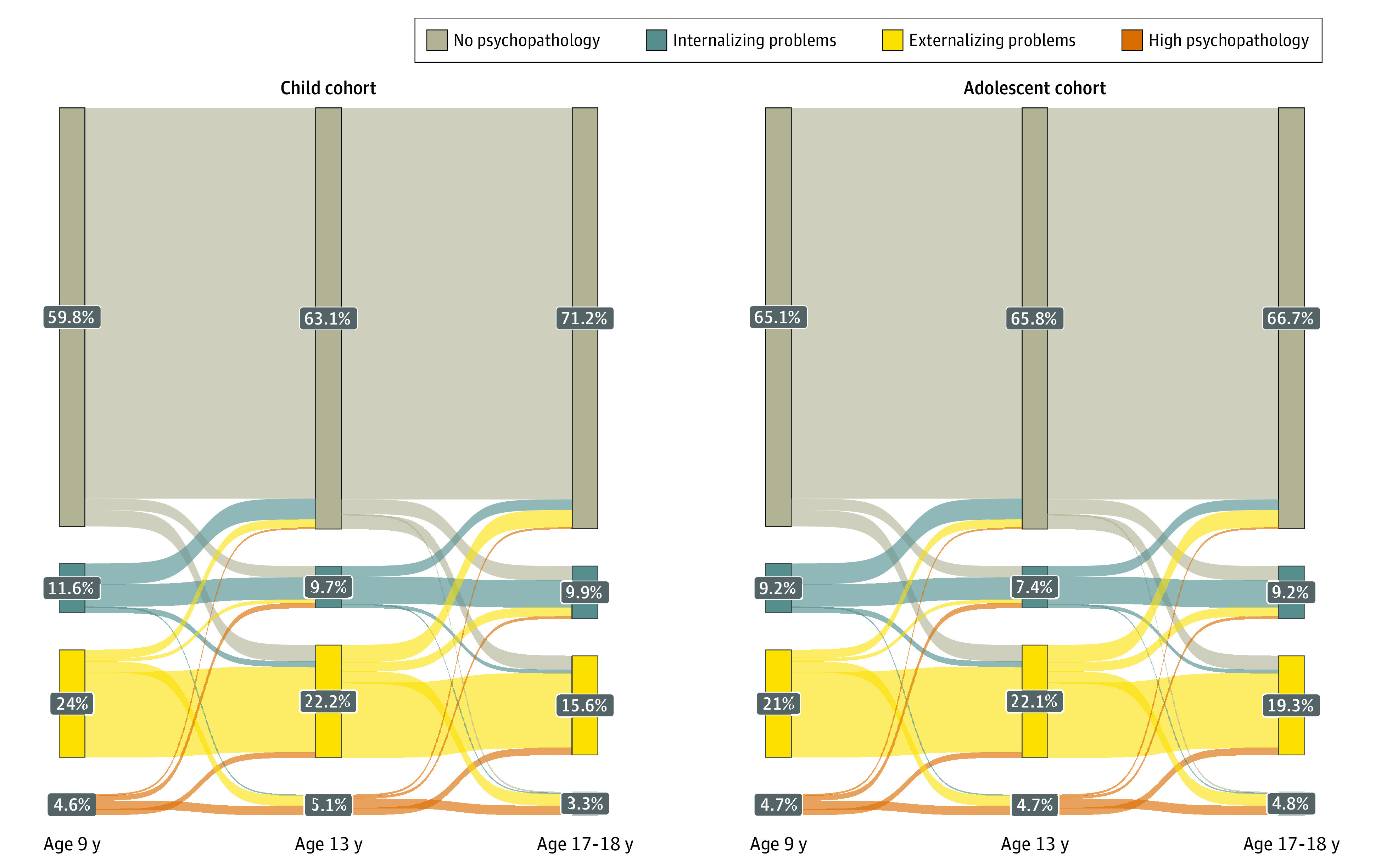
Sankey Diagram Displaying the Transition Between the Waves of the Study in Child Sample and Adolescent Sample

In the child sample, there was cross-wave shifting between all profiles, particularly in the 3 psychopathology profiles (Figure 2; eTables 3, 4, and 7 in the Supplement). Forty-eight percent of participants fell into 1 of the 3 psychopathology profiles at some point between early childhood and late childhood. Approximately 40% to 50% of those with internalizing problems remitted by the following wave of the study (eg, 375 children [43.1%] between wave 1 and wave 2). Only 12% to 30% of those with the externalizing problems profile remitted by the next wave of the study (230 children [12.7%] between wave 1 and wave 2). Similar to the adolescent sample, very few individuals transitioned directly from no psychopathology to high psychopathology (age 3, 27 [0.6%] and age 5, 14 [0.3%]) or high psychopathology to no psychopathology (age 3, 25 [7.2%] and age 5, 17 [4.5%]). Children who presented with new incidents of high psychopathology were previously most often within the externalizing problems profile (age 5, 129 [61.3%] and age 9, 95 [74.3%]). There were several significant differences in the transition percentages between boys and girls at both waves. The overall pattern suggested that in early life a higher percentage of girls had deescalating trajectories relative to boys (eTable 9 in the Supplement).

#### Adolescent Sample

There was a lower rate of transition between the profiles relative to the child sample, but transition was still observed in the 3 psychopathology profiles (ie, high psychopathology, externalizing problems, and internalizing problems). A total of 2661 participants (44.1%) fell into 1 of the 3 psychopathology profiles at some point between childhood and late adolescence (Figure 2; eTable 6 in the Supplement). Twenty-five percent to 42% of those with internalizing problems remitted by the following wave of the study (eg, 230 adolescents [41.5%] between wave 1 and wave 2). Only 8% to 15% of those in the externalizing problems profile remitted by the next wave of the study (eg, 100 adolescents [7.9%] between wave 1 and wave 2). Almost no individuals transitioned directly from no psychopathology to high psychopathology (age 9, 0 and age 13, 10 [0.2%]) and very few from high psychopathology to no psychopathology (age 9, 13 [4.5%] and age 13, 21 [7.3%]). Most new incidents of high psychopathology were preceded by externalizing problems (age 13, 129 [91.1%] and age 17, 146 [89.9%]). There were some (limited) differences in the transition percentage between boys and girls (eTable 8 in the Supplement). Most notably, girls were more likely to transition to the internalizing problem profile from the other 3 profiles relative to boys by age 17 years.

### Persistence of Profiles in the Child Sample and Adolescent Sample

The trajectories of those with persistent psychopathology profiles (ie, remaining in a specific profile over the 3 waves of each cohort) is of potential clinical importance. The percentage of those with persisting psychopathology profiles varied widely across cohorts (Table 2). In the child sample, only 16% to 23% had a form of psychopathology at age 3 years that persisted through to late childhood (internalizing problem, 140 participants [16.2%]; externalizing problem, 407 participants [22.6%]). In the adolescent sample, 20% to 52% of those with a form of psychopathology at age 9 years persisted through to late adolescence (eg, 160 [29%] adolescents with internalizing problems). Of those with no psychopathology at the first wave, 3378 (85.8%) remained so. Between 3% to 4% of all children were in a profile that persisted and was deemed significant as they had subscale scores greater than the 90th percentile (eg, 56 adolescents [0.9%] with high psychopathology and 160 adolescents [2.7%] with internalizing problems). Finally, in the child sample a higher proportion of boys had persistently high psychopathology (eTable 10 in the Supplement) relative to the persistently no psychopathology profile (IRR, 3.74; 95% CI, 2.08-6.74; *P* < .001), while in the adolescent sample a higher distribution of boys had persistent externalizing problems (eTable 11 in the Supplement) relative to the persistently no psychopathology profile (IRR, 1.57; 95% CI, 1.33-1.87; *P* < .001).

**Table 2.** Persistence Profile Membership in the Child Sample and the Adolescent Sample

Profiles	Child cohort (N = 7507)^a^			Adolescent cohort (N = 6039)^b^		
	Classification in wave 1, No.	Same classification in wave 3, No. (%)	Wave 3 same classification, proportion of overall sample, %	Classification in wave 1, No.	Same classification in wave 3, No. (%)	Wave 3 same classification, proportion of overall sample, %
No psychopathology	4487	3856 (85.9)	51.4	3933	3378 (85.9)	55.9
Externalizing problem	1804	407 (22.6)	5.4	1271	658 (51.8)	10.9
Internalizing problem	869	140 (16.2)	1.9	554	160 (29.0)	2.7
High psychopathology	346	63 (18.1)	0.8	282	56 (19.8)	0.9
Any psychopathology	3019	610 (20.2)	8.1	2107	874 (41.4)	14.5
Any persistence	7507	NA	4466 (59.5)	6039	NA	4249 (70.4)

^a^ The child cohort was a persistent class enrolled in 2008 and maintained from early childhood to late childhood.

^b^ The adolescent cohort was a persistent class enrolled in 1998 and maintained from childhood to late adolescence.

### Cross-Cohort Comparison

The full results of the cross-cohort comparison are displayed in supplementary materials (eTable 12 in the Supplement). Briefly, we observed little difference between the profiles, but there were cohort by gender interactions indicating that there was a significantly higher proportion of boys in all 3 psychopathology classes in the child sample relative to the adolescent sample.

## Discussion

Using 2 nationally representative cohorts that spanned from early childhood to late adolescence and person-centered analysis methods, we identified 4 distinct psychopathology profiles for all ages, namely: no psychopathology (approximately 65%), high psychopathology (approximately 5%), externalizing problems (approximately 20%), and internalizing problems (approximately 10%). These groups broadly mirror other studies using latent class analysis to investigate multiple symptoms of psychopathology.^14,15,16,25^

We found that movement between the different profiles was common; between 45% and 50% of young people fell into 1 of the 3 psychopathology categories at some point during development but only 3% to 5% had persistent problems. A similar prevalence of mental disorders is seen in community samples using clinical interviews.^26,27^ This suggests that, for most young people, mental health problems are common but transient phenomena.

Our results complement recent findings that demonstrate the cross-sectional and sequential comorbidity of mental disorders^6,7,8^ and are in line with research emphasizing the fluid nature of psychopathology in youth.^28^ A small proportion of the samples (3% to 4%) had persistent psychopathology, but most of those with psychopathology had remitting or changing trajectories. The identification of early risk factors identifying those with persistent psychopathology is of paramount importance as they are likely to need the most intervention and resources throughout life. Early intervention in these individuals may improve their outcome.

In line with our expectations, there was more fluidity between profiles in early childhood than adolescence. We observed that a greater proportion of children in the externalizing problems profile remained in this profile relative to the internalizing problems profile. By adolescence the vast majority of those who joined the high psychopathology profile had transitioned from the externalizing problems profile (age 13, 91% and age 17, 90%). This suggests that there was a subsample of those in the externalizing problems profile who were vulnerable to escalating problems and may require monitoring. These findings aligned with observations from similar investigations.^15,16^ McElroy and colleagues^15^ found that those with internalizing problems at baseline were more likely to transition to the normative class than those with externalizing problems. They suggest that experiencing early externalizing problems may place an individual at greater risk of cross-domain comorbidity. This is consistent with the high proportion of adults in inpatient psychiatric services^29,30^ and the high service usage across judicial and health care domains^31^ of those with a history of conduct disorder. It also aligns with the observation that those with attention-deficit/hyperactivity disorder (ADHD) in early life are more likely to have concurrent and sequential comorbidity than their peers.^32^ Thus, identifying risk factors for those with externalizing problems and an escalating trajectory, distinguishing them from their peers and intervening early, may assist in reducing the risk of comorbidity and societal health burden and social cost.

In the child sample, boys were more likely to make up a greater proportion of all psychopathology subgroups (eFigure 3 in the Supplement). However, by late adolescence, there were more girls in the internalizing problems profile with more boys in the externalizing problems profile. These trends broadly align with observations from developmental epidemiological samples.^33,34^ The most notable 10-year cross-cohort difference at age 9 years was the higher percentage of boys in the psychopathology profiles in the child sample relative to the adolescent sample. There are several potential explanations for this observation. This could be a cohort effect explained by unmeasured demographic differences between the samples and recruitment strategies or generational differences in those with psychopathology in mid-to-late childhood. To our knowledge, this trend has not been observed elsewhere and requires further investigation.

An important outstanding question is what other risk factors could accurately project to contribute to these profiles and the transition between them? Future research should examine the risk factors and distal outcomes for these profiles and the trajectory between any 2 profiles. Of particular importance is the identification of risk factors for those with persistent psychopathology vs those who remit. Also important will be the identification of risk factors for those with the externalizing problems profile who go on to have high psychopathology.

### Strengths and Limitations

There are a number of strengths to this investigation. First, we used nationally representative data from 2 large longitudinal cohorts spanning across the majority of early life development. In both cohorts the same measures were administered to the respondents allowing for continuity of the findings across cohorts.

This study had several limitations. The SDQ is a widely used measure of psychopathology, but it is limited to the examination of internalizing and externalizing problems. This leaves open the possibility for additional profiles and trajectories if other aspects of psychopathology were measured, for example psychotic experiences and thought disorders.^6^ Moreover, the SDQ was parent report only, which may differ from self-report, particularly in adolescence.^35^

For theoretical reasons we opted to allow time-varying profiles. The rationale for this was that psychopathology is likely to change with time. Thus, we expected that the profile would not be the same at each wave of each study. While the configural model selection in both cohorts at all ages suggested a 4-profile solution, there were degrees of variance in the means of each subscale at the different ages. The additional constraint of time invariance had a significantly poorer fit to the data and is theoretically less plausible given the development period covered in the analyses. Thus, while our profiles are similar across the ages they are not to be interpreted as absolutely identical at each age.

## Conclusions

There is a complex trajectory of psychopathology across youth. The main findings of this study were: (1) mental health problems in youth appear to fall into 3 distinct categories; (2) up to half of all young people had a mental health problem at least once; (3) 90% of new incidence of high psychopathology were preceded by externalizing problems; and (4) roughly 1 in 20 young people had persistent and clinically relevant mental health problems. It is of paramount importance to identify factors that distinguish those with persisting problems and escalating trajectories so that resources can be appropriately directed.

## Notes

Supplement.
eMethods.
**eAppendix.** Supplementary Results **eTable 1.** Technical Reports Accessibility for Missing Data Analysis and Reweighting Procedure **eTable 2.** Pairwise Associations Between the Strengths and Difficulties Questionnaires at Each Wave in 2008 Cohort **eTable 3.** Pairwise Associations Between the Strengths and Difficulties Questionnaires at Each Wave in 1998 Cohort **eTable 4.** Fit Indices, Information Criteria and Entropy for the Latent Profile Analysis and Latent Profile Transition Analysis in the 2008 Cohort at All Ages **eTable 5.** Fit Indices, Information Criteria and Entropy for the Latent Profile Analysis and Latent Profile Transition Analysis in the 1998 Cohort at All Ages **eTable 6.** Transition Percentages in the 1998 Cohort Between Ages 9 and 13 and Ages 13 and 17 **eTable 7.** Transition Percentages in 2008 Cohort Between Ages 3 and 5 and Ages 5 and 9 **eTable 8.** Transition Percentages in Boys and Girls in 1998 Cohort Between Ages 9 and 13 and Ages 13 and 17 **eTable 9.** Transition Percentages in Boys and Girls in the Infant Cohort Between Ages 3 and 5 and Ages 5 and 9 **eTable 10.** Persistence Psychopathology From Early Childhood to Late Childhood in Boys and Girls **eTable 11.** Persistence Psychopathology From Childhood to Late Adolescence in Boys and Girls **eTable 12.** Cohen D Effect-sizes Differences Between the Cohorts and Within Each Profile in the Strengths and Difficulties Questionnaire Subscale Scores at Age 9 **eFigure 1.** BIC and Adjusted BIC Values for the Child and Infant Cohort **eFigure 2.** Fit Indices and Percentage Change in the Fit Indices by Increasing the Profiles in the Latent Transition Analysis for the Child Cohort and Infant Cohort **eFigure 3.** Gender Differences Between the Profiles at Each Age in the Infant and Child Cohorts
eReferences.
