# Error in Figure

**DOI:** 10.1001/jamanetworkopen.2022.27050

**Published:** 2022-07-26

In the Original Investigation titled “Person-Centered Trajectories of Psychopathology From Early Childhood to Late Adolescence,”^1^ published May 10, 2022, a labeling error was corrected in the x-axis of Figure 2; the correct labels for the child cohort are Age 3 y, Age 5 y, and Age 9 y. This article has been corrected.^1^

## References

[1] Healy C, Brannigan R, Dooley N, . Person-centered trajectories of psychopathology from early childhood to late adolescence. JAMA Netw Open. 2022;5(5):e229601. doi:10.1001/jamanetworkopen.2022.9601 35536581 PMC9092205

