# Supplementary material for: Person-Centered Trajectories of Psychopathology From Early Childhood to Late Adolescence
Source: JAMA Netw Open. 2022 May 10;5(5):e229601. doi: 10.1001/jamanetworkopen.2022.9601 (PMC9092205; doi:10.1001/jamanetworkopen.2022.9601)
Supplement: Supplement. — eMethods. eAppendix. Supplementary Results eTable 1. Technical Reports Accessibility for Missing Data Analysis and Reweighting Procedure eTable 2. Pairwise Associations Between the Strengths and Difficulties Questionnaires at Each Wave in 2008 Cohort eTable 3. Pairwise Associations Between the Strengths and Difficulties Questionnaires at Each Wave in 1998 Cohort eTable 4. Fit Indices, Information Criteria and Entropy for the Latent Profile Analysis and Latent Profile Transition Analysis in the 2008 Cohort at All Ages eTable 5. Fit Indices, Information Criteria and Entropy for the Latent Profile Analysis and Latent Profile Transition Analysis in the 1998 Cohort at All Ages eTable 6. Transition Percentages in the 1998 Cohort Between Ages 9 and 13 and Ages 13 and 17 eTable 7. Transition Percentages in 2008 Cohort Between Ages 3 and 5 and Ages 5 and 9 eTable 8. Transition Percentages in Boys and Girls in 1998 Cohort Between Ages 9 and 13 and Ages 13 and 17 eTable 9. Transition Percentages in Boys and Girls in the Infant Cohort Between Ages 3 and 5 and Ages 5 and 9 eTable 10. Persistence Psychopathology From Early Childhood to Late Childhood in Boys and Girls eTable 11. Persistence Psychopathology From Childhood to Late Adolescence in Boys and Girls eTable 12. Cohen D Effect-sizes Differences Between the Cohorts and Within Each Profile in the Strengths and Difficulties Questionnaire Subscale Scores at Age 9 eFigure 1. BIC and Adjusted BIC Values for the Child and Infant Cohort eFigure 2. Fit Indices and Percentage Change in the Fit Indices by Increasing the Profiles in the Latent Transition Analysis for the Child Cohort and Infant Cohort eFigure 3. Gender Differences Between the Profiles at Each Age in the Infant and Child Cohorts eReferences. [file jamanetwopen-e229601-s001.pdf]

Supplemental Online Content

Healy C, Brannigan R, Dooley N, et al. Person-centered trajectories of psychopathology from early childhood to late adolescence. *JAMA Netw Open.* 2022;5(5):e229601. doi:10.1001/jamanetworkopen.2022.9601

eMethods.

eAppendix. Supplementary Results

eTable 1. Technical Reports Accessibility for Missing Data Analysis and Reweighting Procedure

eTable 2. Pairwise Associations Between the Strengths and Difficulties Questionnaires at Each Wave in 2008 Cohort

eTable 3. Pairwise Associations Between the Strengths and Difficulties Questionnaires at Each Wave in 1998 Cohort

eTable 4. Fit Indices, Information Criteria and Entropy for the Latent Profile Analysis and Latent Profile Transition Analysis in the 2008 Cohort at All Ages

eTable 5. Fit Indices, Information Criteria and Entropy for the Latent Profile Analysis and Latent Profile Transition Analysis in the 1998 Cohort at All Ages

eTable 6. Transition Percentages in the 1998 Cohort Between Ages 9 and 13 and Ages 13 and 17

eTable 7. Transition Percentages in 2008 Cohort Between Ages 3 and 5 and Ages 5 and 9

eTable 8. Transition Percentages in Boys and Girls in 1998 Cohort Between Ages 9 and 13 and Ages 13 and 17

eTable 9. Transition Percentages in Boys and Girls in the Infant Cohort Between Ages 3 and 5 and Ages 5 and 9

eTable 10. Persistence Psychopathology From Early Childhood to Late Childhood in Boys and Girls

eTable 11. Persistence Psychopathology From Childhood to Late Adolescence in Boys and Girls

eTable 12. Cohen D Effect-sizes Differences Between the Cohorts and Within Each Profile in the Strengths and Difficulties Questionnaire Subscale Scores at Age 9

© 2022 Healy C et al. *JAMA Network Open.*

eFigure 1. BIC and Adjusted BIC Values for the Child and Infant Cohort

eFigure 2. Fit Indices and Percentage Change in the Fit Indices by Increasing the Profiles in the Latent Transition Analysis for the Child Cohort and Infant Cohort

eFigure 3. Gender Differences Between the Profiles at Each Age in the Infant and Child Cohorts

eReferences.

This supplemental material has been provided by the authors to give readers additional information about their work.

© 2022 Healy C et al. *JAMA Network Open.*

eMethods.

Participants. The Child cohort (Cohort ’08) comprises 11,134 infants born in Ireland in 2007-2008, who were randomly sampled from the child benefit register (a universal monthly social welfare payment). In total, 16,136 families were approached to participate in the 9-month wave 1 of the study and 11,134 agreed. Families from this 11,134 were approached for subsequent follow-ups interviews (with limited exceptions such as emigration, death or refusal) and 9793 agreed at age 3, 9793 at age 5 and 8032 at age 9. The final sample for this investigation included 7,507 families who took part in the study at ages 9 months, 3 years, 5 years and 9 years old. The sample was compared to children under the age of 1 reported in the 2006 Irish census on 11 key variables including family social class, child’s gender, mother’s nationality, family structure. A sampling weight was than applied to Cohort ’08 such that the sample mirrored the 2006 Census on these characteristics. Additionally to maintain representation the sample was reweighted to account for attrition between waves. The final sample was representative of children born in Ireland during this time period and who continued to live in Ireland until at least age 9 (see Thornton et al., 2013).

Missing data comparisons are conducted after each wave of the study (see eTable 1 below for further information). Using this information the sample is reweighted to account for non-participation at the follow up waves. The non-completion weight was combined with the original census weighting to maintain representation.

For the Adolescent sample (Cohort ’98) 910 national primary schools were contacted for participation, of which 82% agreed. Within the participating schools, families of children who

© 2022 Healy C et al. *JAMA Network Open.*

were aged 9 were approached for inclusion and 57% agreed to participate (n=8658). Those who participated at age 9 were contacted for the follow up waves (with limited exceptions) and 7423 agreed at age 13 and 6212 agreed at age 17. For this investigation the final sample includes the 6039 families who took part at ages 9, 13 and 17. Similar to Cohort ’08, this sample was adjusted using a sampling weight to ensure national representation. The sample was compared with the children aged 9 in the 2006 Irish Census based a number of key variables and readjusted for over/under representation. The sample was also reweighted for attrition between the waves. Thus, the final sample was representative of the children living in Ireland who were 9-years-old in 2006 and who continued to live in Ireland until at least age 17/18 years. Missing data comparisons are conducted after each wave of the study (see eTable 1 below for further information). Using this information the sample is reweighted to account for non-participation at the follow up waves. The non-completion weight was combined with the original census weighting to maintain representation.

Strengths and Difficulties Questionnaire additional information. The strengths and difficulties questionnaire consists is a twenty-five item questionnaire measuring prosocial behaviour and four psychopathology sub-scales; emotional symptoms, conduct problems, hyperactivity or inattention, peer problems (Goodman, 1997). Each subscale consists of five items with possible responses including “not true”, “somewhat true” and “certainly true” and scores ranging from zero to two, respectively. Subscale scores were the sum of the scores in each item of the subscale. At each wave, parents/guardians of the participating child/adolescent were required to respond to each item on the questionnaire. The means and standard deviations of each

© 2022 Healy C et al. *JAMA Network Open.*

subscale at each wave are presented in Table 1 and the pairwise linear correlation between the subscale scores at each wave of both cohorts are presented in supplementary eTable 1 and eTable2. There were small to medium significant positive correlations between each of the sub-scales at all waves of the study (r range: 0.22-0.49, all p <.001). The cross-sectional correlations were mostly stable over time (r range difference: 0.01-0.16).

Statistical Analysis. Analyses were carried out in MPLUS (Muthén & Muthén, 2009) and Stata (Statacorp, 2017). Both cohorts were analysed in the same way and both were weighted for national representation. The demographic characteristics of each cohort are presented in Table 1. Latent profile transition analysis involved exploratory latent profile analysis at each age of both cohorts (Child sample: 3, 5, 9 years; and Adolescent sample: 9, 13 and 17 years) followed by latent profile transition analysis as per Masyn (2013). Latent profile transition analysis allows for examination changes in identified subgroups of a latent construct (in this case psychopathology) over time based on estimated probabilities of subgroup membership at each time point and the estimated probability of transition between the subgroups over time. Within this analysis these models were estimated with profile invariant diagonal variance-covariance matrix structure such that variance for the same construct (i.e. emotional problems) are constrained to be equal across profiles and residual covariance's were set to zero (Johnson, 2021). This prevented profile items from co-varying above there conditional profile membership. Nylund Asparouhov & Muthén (2007) recommend the use bayesian information criteria and the bootstrapped likelihood ratio test to indicate correct class selection based on monte carlo simulation studies. Due to the representative weighting, it is not possible to

calculate a bootstrapped likelihood ratio test. Thus, the optimum number of profiles were determined based on bayesian information criteria and adjusted-bayesian information criteria as well as profile interpretability. Global information criteria do not always arrive at a single lowest value. Thus, in line with Masyn's (2013) suggestion, the optimum model fit was determined based on the diminishing gains in model fit (elbow). The elbow in the model fit metrics refers a point of inflection in the model fit gains. This is reflected by a levelling-off in the improvement in the model fit with the addition of another profile, such that adding the additional profile only marginally improves the fit to the data relative to the improvement seen by the addition of previous profile. Visually this point resembles an elbow in the model fit data points. The global fit metrics were independently reviewed by two of the authors (CH and LS). Model selection is a parsimonious procedure such that including an additional profile should require notable improve model fit. Examining for an elbow in the fit metrics can assist with model selection. This may be particularly important in the case of latent profile analysis where the items are continuous and thus have a greater quantity of information than latent class analysis where items are binary. Thus, within latent profile analysis there may not always be a clear point at which the model fit stops improving with additional profiles and so the diminishing gains in model fit is used to guide the process via a search for the elbow in the model fit metrics. We examined the number of profiles, and the prevalence of each profile and its transition period. Given the duration and significance of the developmental period being investigated, it is conceivable that the mean and variance of the items loading to each profile may differ across this period and thus the profiles were allowed to vary with time. In addition to these theoretical reason this was statistically examined using Bayesian information criteria, a -

© 2022 Healy C et al. *JAMA Network Open.*

2log likelihood ratio test for nested models (see https://www.statmodel.com/chidiff.shtml) and global classification accuracy as assessed by entropy. The profile labelling was based on the mean and variance of the scores in the SDQ. For ease of interpretability this is presented in Figure 1 and 2 in percentiles. Profile assignment was based on the highest designated posterior probability.

Multinomial logistic regression was conducted to examine potential differences in sex distribution between the profiles, transitions and in the persistence of the profile. The results are reported as Incidence Risk Ratios (IRR). This was conducted as an auxiliary analysis based on the profile assignment at each age. These analyses were also weighted for national representation and attrition within the sample.

We conducted a cross-cohort comparison at age 9 years. We compared the prevalence of the profiles and discrepancies in sex distribution of the profiles using multinomial logistic regression. These analysis were adjusted for demographic differences between the sample including primary care givers highest level of education, nationality of the child and nationality of the . We also report the effect sizes (Cohens d) for differences in the interpretation of the profiles between the cohorts at age 9 years.

© 2022 Healy C et al. *JAMA Network Open.*

eAppendix. Supplementary Results

Comparison between the 3-profile, 4-profile and 5-profile solution. Within the infant cohort we also considered the 3-profile and 5-profile solution. The rationale operated on two levels; overall model fit and profile distinction (informed by the literature). The 4-profile solution had better model fit metrics to the 3-profile solution and model fit gains were not diminished with the addition of the 4-profile solution (eFigure 2b and d). The 3-profile solution did distinguishes between high all psychopathology and low psychopathology, similar to the 4-profile solution. The third profile however had limited distinction from the high psychopathology class at age 3 and 5. By age 9 it fails to distinguish an identifiable subgroup and merely reflects a "middle level difficulties" across the subscales. The 5-profile solution had better model fit then the 4-profile solution (lower BIC) however, it had notably diminished model gains (relative to the 3-4 profile change gains (eFigure 2b and d). This was indicative of an elbow at the 4-profile solution. Additionally the 5-profile solution was similar to the 4-profile solution re-identifying the same four profiles. The additional profile was very similar to the high psychopathology profile and did not have clear distinguishing features. The lack of distinction added to our decision that a four-profile solution was optimal and aligned better with the literature.

Time varying and Time invariant comparison. We compared the time varying and time invariant models of the child and adolescent cohorts. In all analyses the time varying model had better performance with lower Bayesian information criteria (Child sample: Time Varying = 332182.67; Time Invariant = 335003.73; and Adolescent Sample: Time Varying = 268568.60; Time Invariant = 335003.73), significantly better model fit based on the log-likelihood ratio test (Child Sample: X^2 (32) = 717.57, p <.001; Adolescent Sample: X^2 (32) = 201.94, p <.001), and

slight better classification accuracy (Child sample: Time Varying = 0.85; Time Invariant = 0.83; and Adolescent Sample: Time Varying = 0.87; Time Invariant = 0.86). For these and the theoretical reasons of how psychopathology changes across development, the time varying model was used.

Cross-Cohort Comparison. Both samples measured children at age 9 using the same psychopathology measure allowing for a 10 years cross-cohort comparison. There were few meaningful differences in mean SDQ scores in each profile between the cohorts. The exception was elevated peer and hyperactivity problems in the High Psychopathology profile of the Child sample (medium effect sizes). All other between-cohort differences in SDQ scores were small (absolute range: 0.03-0.31, see Supplementary Table 9). The percentage of each profile at age 9 in each samples were broadly similar. Multinomial logistic regression examining the effects of cohort, sex and their interaction revealed that the Adolescent sample had significantly greater percentage of children with Externalising Problems (IRR:1.75, CI:1.53-2.01, p<.001), High Psychopathology (IRR:2.25, CI:1.69-2.99, p<.001) and Internalising Problems (IRR:1.21, CI:1.02-1.43, p=.02) when compared with the Child sample. At age 9 irrespective of the sample, males were significantly more likely to have Externalising Problems (IRR:1.80, CI:1.58-2.05, p<.001), High Psychopathology (IRR:2.34, CI:1.78-3.08, p<.001) and Internalising Problems (IRR:1.31, CI:1.12-1.52, p=.001). There were cohort by sex interactions which indicated that there were significantly higher proportion of males in all three psychopathology classes in the Child sample relative to the Adolescent sample (High Psychopathology: IRR:1.87, CI:1.30-2.69, p <.001; Externalising Problems: IRR:1.34, CI:1.12-1.62, p=.001; and Internalising Problems: IRR:1.43, CI:1.13-1.81, p=.003).

© 2022 Healy C et al. *JAMA Network Open.*

eTable 1. Technical Reports Accessibility for Missing Data Analysis and Reweighting Procedure

	Section and Table	Reference
Cohort 08’		
Age 9 Months	Section 2.6<br>Table 2.5 and 2.6	Thornton, M., Williams, J., McCrory, C., Murray, A., Quail, A., (2013) Growing Up in Ireland: Design, Instrumentation and Procedures for the Infant Cohort at Wave 1 (9 months). Infant Cohort Technical Report No. 2. Dublin: Department of Children and Youth Affairs
Age 3 Years	Section 2.2 and 2.3<br>Table 2.5.	McCrory, C., Williams, J., Murray, A., Quail, A., Thornton, M. (2013) Growing Up in Ireland: Design, Instrumentation and Procedures for the Infant Cohort at Wave 2 (3 years). Infant Cohort Technical Report No. 3. Dublin: Department of Children and Youth Affairs
Age 5 Years	Section 2.5<br>Table 2.4, 2.5 and 2.6.	Williams, J., Thornton, M., Murray, A., Quail, A. (2019) Growing Up in Ireland: Design, Instrumentation and Procedures for the Infant Cohort at Wave 3 (5 years). Infant Cohort Technical Report No. 4. Dublin: Department of Children and Youth Affairs
Age 9 Years	Section 2.8<br>Table 2.8.	McNamara, E., O’ Mahony, D., Murray, A., Quail, A. (2020) Growing Up in Ireland: Design, Instrumentation and Procedures for the Infant Cohort at Wave 5 (9 years). Infant Cohort Technical Series No. 20. Dublin: Department of Children and Youth Affairs

© 2022 Healy C et al. *JAMA Network Open.*

Cohort 98’		
Age 9 Years	Section 2.2 and 2.4.	Murray, A., McCrory, C., Thornton, M., Williams, J., Quail, A., Swords, L., Doyle E., Harris E. (2009) Growing Up in Ireland: Design, Instrumentation and Procedures for the Child Cohort at Wave 1 (9 years). Child Cohort Research Report No. 1. Dublin: Department of Children and Youth Affairs
Age 13 Years	Section 2.5-2.6<br>Table 2.2 and 2.3	Thornton, M., Williams, J., McCrory, C., Murray, A., Quail, A., (2016) Growing Up in Ireland: Design, Instrumentation and Procedures for the Child Cohort at Wave 2 (13 years). Child Cohort Technical Report No. 2. Dublin: Department of Children and Youth Affairs
Age 17/18 Years	Section 2.3<br>Table 2.3 and 2.4.	Murphy, D., Williams, J., Murray, A., Smyth, E., (2016) Growing Up in Ireland: Design, Instrumentation and Procedures for the Child Cohort at Wave 3 (17/18 years). Child Cohort technical Report Series No. 2019-5. Dublin: Department of Children and Youth Affairs

Note: All reports are available at https://www.growingup.ie/data-documentation/

© 2022 Healy C et al. *JAMA Network Open.*

eTable 2. Pairwise Associations Between the Strengths and Difficulties Questionnaires at Each Wave in 2008 Cohort

	**Strength and Difficulty Questionnaire Subscales**			
	Emotional Problems	Peer Problems	Conduct Problems	Hyperactivity/Attention
**Strength and Difficulty Questionnaire Subscales Age 3**				
Emotional Problems	-	-	-	-
Peer Problems	0.25	-	-	-
Conduct Problems	0.24	0.22	-	-
Hyperactivity/Attention	0.21	0.22	0.42	-
**Strength and Difficulty Questionnaire Subscales Age 5**				
Emotional Problems	-	-	-	-
Peer Problems	0.34	-	-	-
Conduct Problems	0.28	0.29	-	-
Hyperactivity/Attention	0.24	0.28	0.47	-
**Strength and Difficulty Questionnaire Subscales Age 9**				
Emotional Problems	-	-	-	-
Peer Problems	0.43	-	-	-
Conduct Problems	0.35	0.34	-	-
Hyperactivity/Attention	0.33	0.36	0.48	-

Note: all analysis were significant to $p<.001$

© 2022 Healy C et al. *JAMA Network Open.*

eTable 3. Pairwise Associations Between the Strengths and Difficulties Questionnaires at Each Wave in 1998 Cohort

	**Strength and Difficulty Questionnaire Subscales**			
	Emotional Problems	Peer Problems	Conduct Problems	Hyperactivity/Attention
**Strength and Difficulty Questionnaire Subscales Age 9**				
Emotional Problems	-	-	-	-
Peer Problems	0.41	-	-	-
Conduct Problems	0.29	0.30	-	-
Hyperactivity/Attention	0.22	0.25	0.42	-
**Strength and Difficulty Questionnaire Subscales Age 13**				
Emotional Problems	-	-	-	-
Peer Problems	0.40	-	-	-
Conduct Problems	0.35	0.23	-	-
Hyperactivity/Attention	0.33	0.24	0.48	-
**Strength and Difficulty Questionnaire Subscales Age 17**				
Emotional Problems	-	-	-	-
Peer Problems	0.38	-	-	-
Conduct Problems	0.30	0.23	-	-
Hyperactivity/Attention	0.31	0.22	0.49	-

Note: all analysis were significant to p<.001

© 2022 Healy C et al. *JAMA Network Open.*

eTable 4. Fit Indices, Information Criteria and Entropy for the Latent Profile Analysis and Latent Profile Transition Analysis in the 2008 Cohort at All Ages

Model	Parameters	BIC	Adj-BIC	AIC	Log likelihood	Entropy
Age 3						
2 Profile	13	113454.09	113412.77	113364.08	-56669.039	0.754
3 Profile	18	112149.93	112092.73	112025.35	-55994.673	0.794
4 Profile	23	111497.54	111424.45	111338.34	-55646.171	0.815
5 Profile	28	110875.27	110786.3	110681.47	-55312.737	0.801
6 Profile	33	110488.08	110383.21	110259.67	-55096.834	0.803
7 Profile	38	110176.29	110055.53	109913.27	-54918.636	0.807
8 Profile	43	109575.25	109438.6	109277.63	-54595.813	0.85
Age 5						
2 Profile	13	113687.83	113646.52	113597.82	-56785.91	0.855
3 Profile	18	112106.28	112049.08	111981.66	-55972.828	0.814
4 Profile	23	111160.18	111087.09	111000.93	-55477.467	0.834
5 Profile	28	110396.41	110307.44	110202.55	-55073.277	0.848
6 Profile	33	110086.91	109982.05	109858.43	-54896.217	0.83
7 Profile	38	109474.56	109353.81	109211.47	-54567.734	0.837
8 Profile	43	109320.92	109184.28	109023.21	-54468.604	0.818
Age 9						
2 Profile	13	116541.83	116500.52	116451.83	-58212.913	0.883
3 Profile	18	115017.67	114960.47	114893.04	-57428.521	0.869
4 Profile	23	113690.95	113617.87	113531.71	-56742.856	0.89
5 Profile	28	112942.29	112853.31	112748.43	-56346.216	0.88
6 Profile	33	112486.39	112381.52	112257.91	-56095.955	0.858
7 Profile	38	111745.13	111624.37	111482.03	-55703.014	0.877
8 Profile	43	111475.35	111338.7	111177.63	-55545.817	0.865
Latent Profile Transition analysis (unconstrained)						
2 Profile	41	340463.89	340333.6	340180.027	-170049.014	0.84
3 Profile	62	335481.87	335284.8	335052.606	-167464.303	0.842
4 Profile	87	332182.67	331906.2	331580.322	-165703.161	0.851
5 Profile	116	329800.12	329431.5	328996.987	-164382.493	0.848
6 Profile	149	327944.22	327470.7	326912.609	-163307.305	0.847
Latent Profile Transition analysis (constrained)						
2 Profile	25	343778.962	343699.5	343605.9	-171777.936	0.836
3 Profile	38	338448.92	338328.2	338185.8	-169054.912	0.844
4 Profile	55	335003.73	334829.0	334622.9	-167256.464	0.833
5 Profile	76	332584.63	332343.1	332058.4	-165953.219	0.851
6 Profile	101	330528.235	330207.3	329829	-164813.476	0.851

© 2022 Healy C et al. *JAMA Network Open.*

eTable 5. Fit Indices, Information Criteria and Entropy for the Latent Profile Analysis and Latent Profile Transition Analysis in the 1998 Cohort at All Ages

Model	Parameters	BIC	Adj-BIC	AIC	Log likelihood	Entropy
Age 9						
2 Profile	13	94493.486	94452.176	94406.308	-47190.154	0.905
3 Profile	18	93389.996	93332.797	93269.289	-46616.644	0.86
4 Profile	23	92446.632	92373.545	92292.395	-46123.197	0.874
5 Profile	28	92007.036	91918.059	91819.268	-45881.634	0.863
6 Profile	33	91320.276	91619.032	91502.599	-45718.299	0.849
7 Profile	38	91015.025	91199.522	91065.448	-45494.724	0.844
8 Profile	43	91065.775	90929.133	90777.417	-45345.709	0.851
Age 13						
2 Profile	13	93299.283	93257.973	93212.105	-46593.053	0.866
3 Profile	18	91956.816	91899.617	91836.108	-45900.054	0.902
4 Profile	23	90758.967	90685.88	90604.729	-45279.365	0.87
5 Profile	28	90194.809	90105.833	90007.041	-44975.52	0.864
6 Profile	33	89751.176	89646.312	89529.879	-44731.939	0.876
7 Profile	38	89282.866	89162.113	89028.038	-44476.019	0.887
8 Profile	43	88897.305	88760.663	88608.947	-44261.474	0.879
Age 17						
2 Profile	13	91660.056	91618.746	91572.878	-45773.439	0.867
3 Profile	18	90377.534	90320.335	90256.826	-45110.413	0.88
4 Profile	23	89430.341	89357.253	89276.103	-44615.052	0.848
5 Profile	28	88941.418	88852.441	88753.65	-44348.825	0.853
6 Profile	33	88598.282	88493.418	88376.985	-44155.492	0.849
7 Profile	38	88319.184	88198.43	88064.356	-43994.178	0.857
8 Profile	43	87789.248	87652.606	87500.89	-43707.445	0.878
Latent Profile Transition analysis (unconstrained)						
2 Profile	41	276764.45	276634.2	276489.5	-138203.754	0.879
3 Profile	62	272519.32	272322.3	272103.6	-135989.776	0.884
4 Profile	87	268568.6	268292.1	267985.2	-133905.59	0.867
5 Profile	116	266877.62	266509	266099.7	-132933.864	0.86
6 Profile	149	265421.9	264948.4	264422.7	-132062.355	0.864
Latent Profile Transition analysis (constrained)						
2 Profile	25	277409.575	277330.1	277241.9	-138595.963	0.873
3 Profile	38	273240.951	273120.2	272986.1	-136455.062	0.872
4 Profile	55	269192.707	269017.9	268823.9	-134356.939	0.861
5 Profile	76	267546.955	267305.5	267037.3	-133442.65	0.86
6 Profile	101	265922.697	265601.8	265245.4	-132521.696	0.857

© 2022 Healy C et al. *JAMA Network Open.*

eTable 6. Transition Percentages in the 1998 Cohort Between Ages 9 and 13 and Ages 13 and 17

Age 9	Age 13			
	No Psychopathology (n=3976)	High all Psychopathology (n=285)	Externalizing Problems (n=1334)	Internalizing problems only (n=444)
No Psychopathology (n=3933)	92.4	0	4.7	2.9
High all Psychopathology (n=282)	4.5	50.8	26.6	18.1
Externalizing Problems (N=1271)	7.9	10.1	78.7	3.3
Internalizing problems only (n=554)	41.5	2.3	13.4	42.8
Age 13	Age 17			
	No Psychopathology (n=4030)	High all Psychopathology (n=291)	Externalizing Problems (n=1164)	Internalizing problems only (n=554)
No Psychopathology (n=3976)	92.8	0.2	3.8	3.2
High all Psychopathology (n=285)	7.3	45.2	35.5	12.0
Externalizing Problems (n=1334)	15.4	10.9	65	8.87
Internalizing problems only (n=444)	25.5	1.5	10.3	62.7

© 2022 Healy C et al. *JAMA Network Open.*

eTable 7. Transition Percentages in 2008 Cohort Between Ages 3 and 5 and Ages 5 and 9

Age 3	Age 5			
	No Psychopathology (n=4734)	High all Psychopathology (n=382)	Externalizing Problems (n=1663)	Internalizing problems only (n=729)
No Psychopathology (n=4487)	91.5	0.6	3.1	4.9
High all Psychopathology (n=346)	7.2	49.4	31.9	11.6
Externalizing Problems (N=1804)	12.7	7.2	72.0	8.1
Internalizing problems only (n=869)	43.1	6.3	13.2	37.4
Age 5	Age 9			
	No Psychopathology (n=5347)	High all Psychopathology (n=249)	Externalizing Problems (n=1167)	Internalizing problems only (n=744)
No Psychopathology (n=4734)	93.7	0.3	1.3	4.6
High all Psychopathology (n=382)	4.5	31.6	39.3	24.5
Externalizing Problems (n=1663)	30.8	5.7	55.4	8.1
Internalizing problems only (n=729)	52.2	2.6	4.6	40.7

© 2022 Healy C et al. *JAMA Network Open.*

eTable 8. Transition Percentages in Boys and Girls in 1998 Cohort Between Ages 9 and 13 and Ages 13 and 17

Age 9			Age 13							
			No Psychopathology (n=3976)		High all Psychopathology (n=285)		Externalizing Problems (n=1334)		Internalizing problems only (n=444)	
	Boys	Girls	Boys	Girls	Boys	Girls	Boys	Girls	Boys	Girls
No Psychopathology (n=3933)	49.5	50.5	92.2	92.6	0.0	0.0	4.9	4.5	3.0	2.9
High all Psychopathology (n=282)	55.2	44.9	3.7	5.6	51.2	50.3	30.6	21.6	14.5	22.6
Externalizing Problems (N=1271)	56.7	43.3	7.7	8.2	10.3	10.0	77.9	79.8	4.1	2.1
Internalizing problems only (n=554)	47.2	52.8	43.0	40.1	2.2	2.4	**9.1****	**17.3****	45.8	40.2
**Age 13**			**Age 17**							
			No Psychopathology (n=4030)		High all Psychopathology (n=291)		Externalizing Problems (n=1164)		Internalizing problems only (n=554)	
	Boys	Girls	Boys	Girls	Boys	Girls	Boys	Girls	Boys	Girls
No Psychopathology (n=3976)	49.5	50.6	94.1	91.6	0.3	0.2	4.2	3.4	**1.5*****	**4.8*****
High all Psychopathology (n=285)	55.9	44.1	9.0	5.2	49.5	39.7	33.9	37.6	**7.6****	**17.6****
Externalizing Problems (n=1334)	54.5	45.5	15.8	14.9	**9.4****	**12.7****	71.1	57.7	**3.7*****	**14.7*****
Internalizing problems only (n=444)	51.7	48.3	26.4	24.6	2.2	0.9	12.6	7.8	58.8	66.8

Note: Emboldened values denote a significant difference between boys and girls in the transition percentages. All comparisons are made relative to remaining in the same group at the following wave. *: p <.05; **: p<.01; and *** p <.001.

© 2022 Healy C et al. *JAMA Network Open.*

eTable 9. Transition Percentages in Boys and Girls in the Infant Cohort Between Ages 3 and 5 and Ages 5 and 9

Age 3			Age 5							
			No Psychopathology (n=4734)		High all Psychopathology (n=382)		Externalizing Problems (n=1663)		Internalizing problems only (n=729)	
	Boys	Girls	Boys	Girls	Boys	Girls	Boys	Girls	Boys	Girls
No Psychopathology (n=4487)	47.8	52.2	90.6	92.2	0.5	0.7	**3.9****	**2.4****	5.0	4.7
High all Psychopathology (n=346)	61.7	38.2	**5.0***	**10.8***	54.0	41.9	32.4	31.1	**8.7***	**16.2***
Externalizing Problems (N=1804)	54.7	45.3	**10.8****	**15.1****	8.4	5.7	74.3	69.2	**6.6****	**9.9****
Internalizing problems only (n=869)	58.1	42.0	38.4	49.7	**7.8***	**4.1***	**17.2*****	**7.8*****	36.6	38.4
**Age 5**			**Age 9**							
			No Psychopathology (n=5347)		High all Psychopathology (n=249)		Externalizing Problems (n=1167)		Internalizing problems only (n=744)	
	Boys	Girls	Boys	Girls	Boys	Girls	Boys	Girls	Boys	Girls
No Psychopathology (n=4734)	47.7	52.3	92.4	95.0	**0.5***	**0.1***	1.6	1.1	**5.5****	**3.8****
High all Psychopathology (n=382)	64.8	35.2	**3.8***	**5.9***	39.3	17.6	**35.0*****	**47.3*****	**21.9*****	**29.4*****
Externalizing Problems (n=1663)	58.5	41.5	**27.1*****	**36.0*****	5.4	6.1	59.6	49.6	7.9	8.3
Internalizing problems only (n=729)	51.7	48.3	51.7	52.7	2.1	3.1	**6.6****	**2.4****	39.6	41.8

Note: Emboldened values denote a significant difference between boys and girls in the transition percentages. All comparisons are made relative to remaining in the same group at the following wave. *: p <.05; **: p<.01; and *** p <.001.

© 2022 Healy C et al. *JAMA Network Open.*

eTable 10. Persistence Psychopathology From Early Childhood to Late Childhood in Boys and Girls

	Persistent class maintained in Cohort 08’											
Classes	No Psychopathology		Externalizing Problems		Internalizing problems only		High all Psychopathology		Any Psychopathology		Any	
	Boys	Girls	Boys	Girls	Boys	Girls	Boys	Girls	Boys	Girls	Boys	Girls
N at age 3 only	2146	2341	987	817	505	365	214	132	1706	1314	3852	3654
% within that class	83.9	87.9	20.8	24.7	14.7	18.1	22.4	11.3	19.2	21.5	-	-
% of the sample	**46.7*** **	**56.3*** **	5.3	5.5	1.9	1.8	**1.2*** **	**0.4*** **	8.5	7.7	55.2	64.0
N Persistence	1800	2057	205	202	74	66	48	15	327	283	2127	2340

Note: Emboldened values denote a significant difference between boys and girls in the persistence percentages. All variables are dummy coded, thus comparisons are relative to not persisting in that group. *: p <.05; **: p<.01; and *** p <.001.

© 2022 Healy C et al. *JAMA Network Open.*

eTable 11. Persistence Psychopathology From Childhood to Late Adolescence in Boys and Girls

	Persistent class maintained in Cohort 98'											
Classes	No Psychopathology		Externalizing Problems		Internalizing problems only		High all Psychopathology		Any Psychopathology		Any	
	Boys	Girls	Boys	Girls	Boys	Girls	Boys	Girls	Boys	Girls	Boys	Girls
N at age 9 only	1945	1987	720	550	261	292	156	126	1137	968	3082	2957
% within that class	87.0	84.9	56.0	46.4	29.1	28.8	34.2	37.3	46.8	39.9	-	-
% of the sample	54.9	57.1	**13.1*** **	**8.6*** **	2.5	2.8	1.7	1.6	17.3	13.1	72.2	70.1
N Persistence	1692	1687	403	255	76	84	53	47	532	386	2224	2073

Note: Emboldened values denote a significant difference between boys and girls in the persistence percentages. All variables are dummy coded, thus comparisons are relative to not persisting in that group. *: p <.05; **: p<.01; and *** p <.001.

© 2022 Healy C et al. *JAMA Network Open.*

eTable 12. Cohen D Effect-sizes Differences Between the Cohorts and Within Each Profile in the Strengths and Difficulties Questionnaire Subscale Scores at Age 9

**Profile**	**Strengths and Difficulties Questionnaire Subscales**			
	Emotional	Peer	Conduct	Hyper
No psychopathology	-0.06<br>(-0.11 – -0.03)	-0.15<br>(-0.19 – -0.11)	-0.07<br>(-0.11 – -0.03)	0.11<br>(0.07 – 0.15)
Externalising problems	0.31<br>(0.23 – 0.40)	0.19<br>(0.11 – 0.28)	0.20<br>(0.12 – 0.29)	0.30<br>(0.21 – 0.38)
Internalising problems only	-0.03<br>(-0.14 – 0.09)	-0.15<br>(-0.27 – -0.04)	0.20<br>(0.08 – 0.32)	0.28<br>(0.17 – 0.40)
High Psychopathology	0.08<br>(-0.12 – 0.27)	0.61<br>(0.41 – 0.81)	-0.26<br>(-0.45 – -0.06)	0.62<br>(0.42 – 0.82)
Total Cohort Differences	-0.03<br>(-0.06 – 0.01)	-0.06<br>(-0.10 – -0.03)	-0.08<br>(-0.11 – 0.04)	0.05<br>(-0.02 – 0.09)

eFigure 1. BIC and Adjusted BIC Values for the Child and Infant Cohort

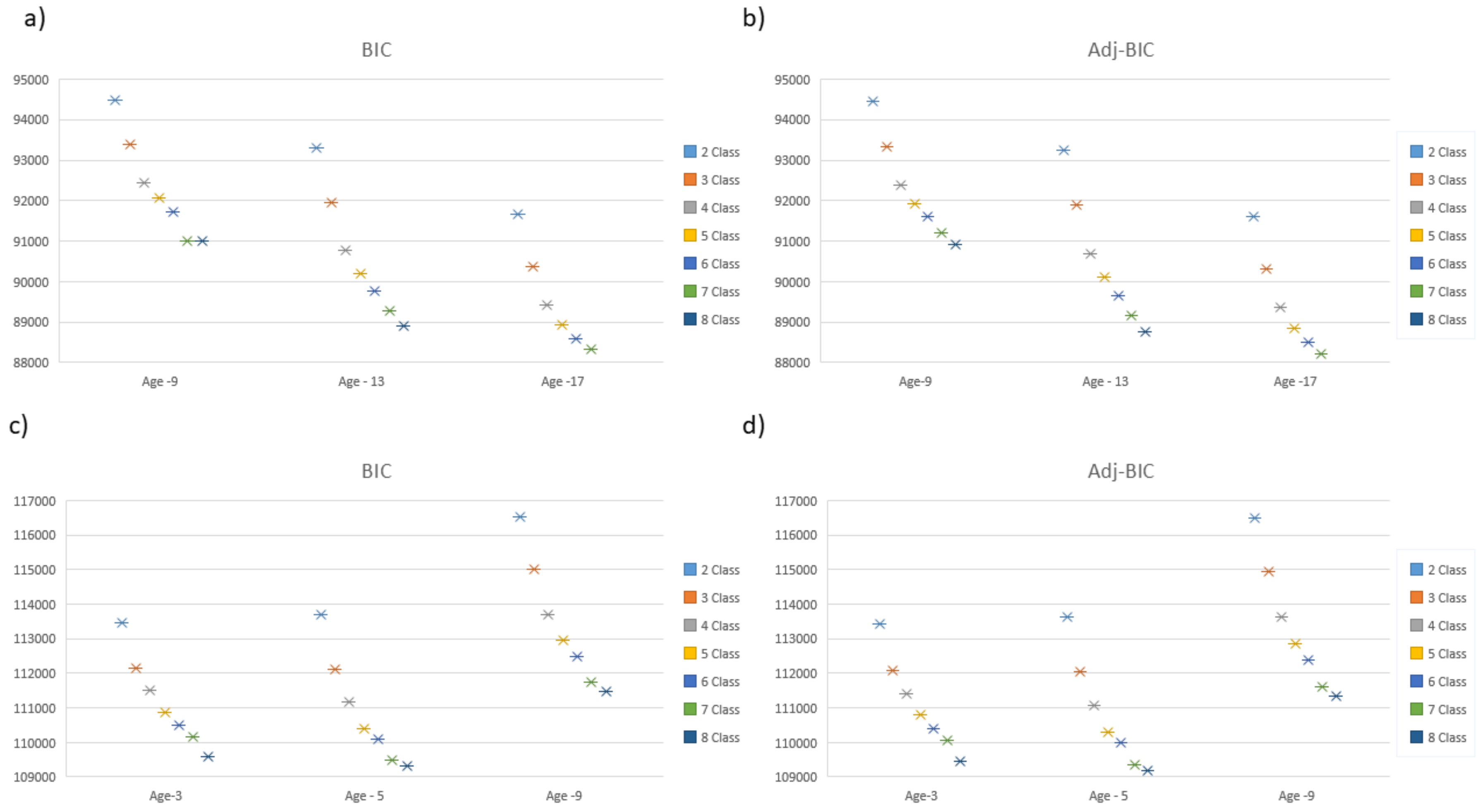

A, BIC for the child cohort; B, Adjusted BIC for the child cohort; C, BIC for the infant cohort; D, Adjusted BIC for the infant cohort.

© 2022 Healy C et al. *JAMA Network Open.*

eFigure 2. Fit Indices and Percentage Change in the Fit Indices by Increasing the Profiles in the Latent Transition Analysis for the Child Cohort and Infant Cohort

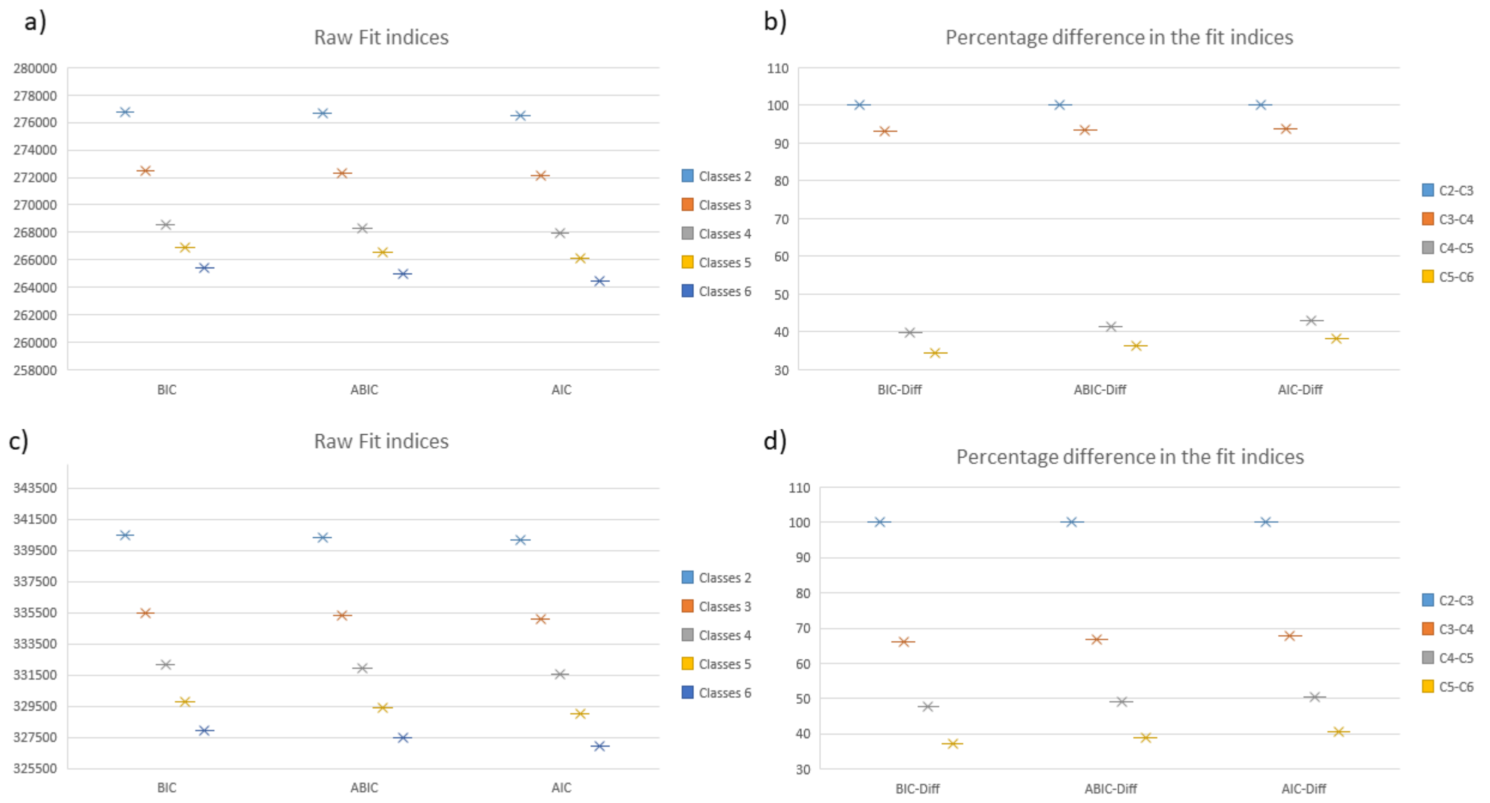

A, fit indices for the child cohort; B, percentage change in fit indices for the child cohort; C, fit indices for the infant cohort; D, percentage change in fit indices for the infant cohort. Note: The percentage change is relative to the change from profile 2 to profile 3 (represent as 100%).

© 2022 Healy C et al. *JAMA Network Open.*

eFigure 3. Gender Differences Between the Profiles at Each Age in the Infant and Child Cohorts

Top row: infant cohort; bottom row: child cohort.

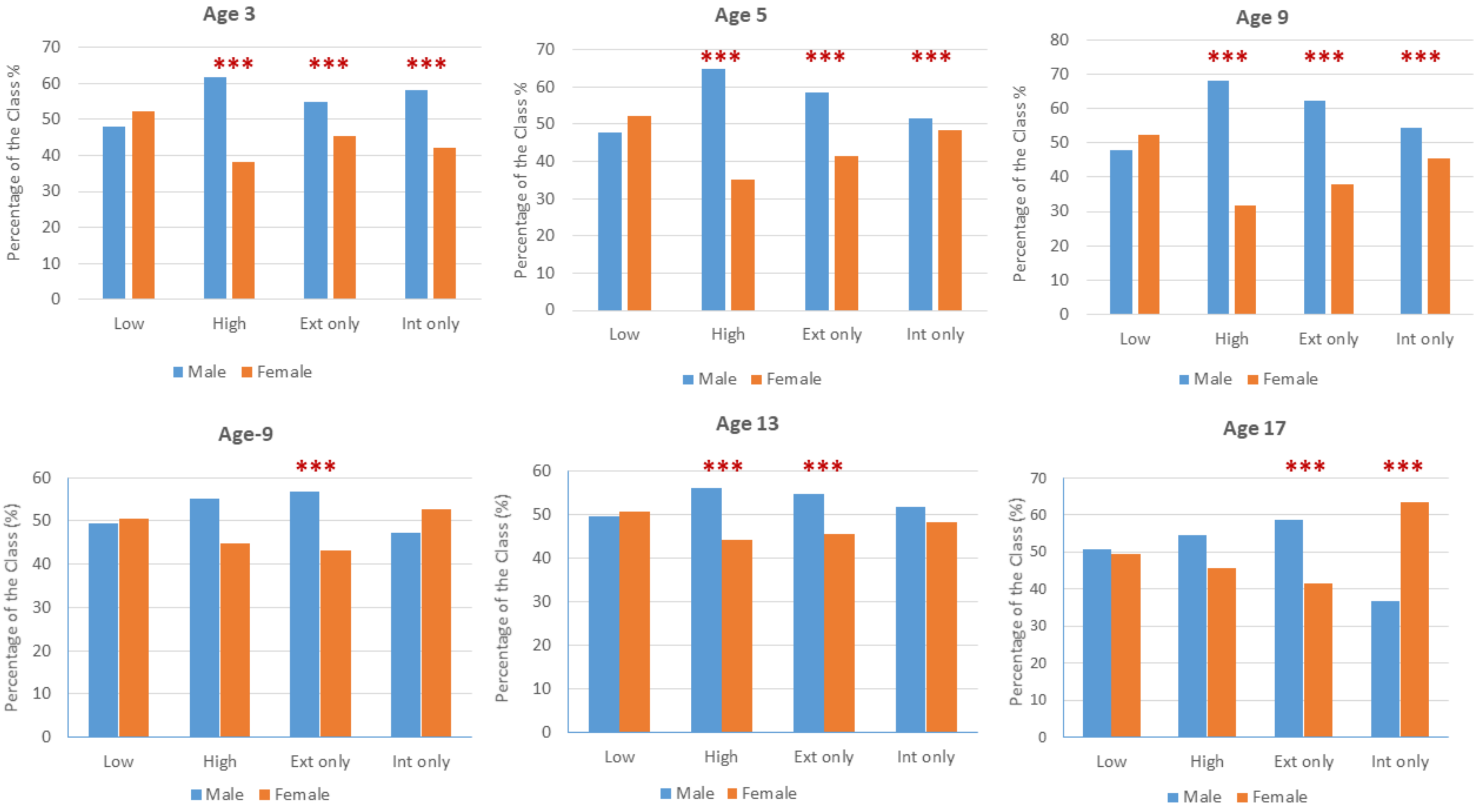

© 2022 Healy C et al. *JAMA Network Open.*

eReferences

Thornton, M., Williams, J., McCrory, C., Murray, A., Quail, A., (2016) Growing Up in Ireland: Design, Instrumentation and Procedures for the Child Cohort at Wave 2 (13 years). Child Cohort Technical Report No. 2. Dublin: Department of Children and Youth Affairs

Goodman, R. (1997). The Strengths and Difficulties Questionnaire: a research note. Journal of child psychology and psychiatry, 38(5), 581-586.

Muthén LK & Muthén BO. Mplus. (2009). Statistical analysis with latent variables. User's guide, 7.

StataCorp. Stata Statistical Software: Release 15. (2017). College Station, TX: StataCorp LLC.

Masyn., K.E. (2013). Latent class analysis and finite mixture modeling. In TD Little (Ed.), Oxford library of psychology. The Oxford handbook of quantitative methods: Statistical analysis. 551–611. Oxford University Press.

Nylund, K. L., Asparouhov, T., & Muthén, B. O. (2007). Deciding on the number of classes in latent class analysis and growth mixture modeling: A Monte Carlo simulation study. *Structural equation modeling: A multidisciplinary Journal*, *14*(4), 535-569.

© 2022 Healy C et al. *JAMA Network Open.*
